# Supplementary material for: Fine-scale collective movements reveal present, past and future dynamics of a multilevel society in Przewalski’s horses
Source: Nat Commun. 2023 Sep 5;14:5096. doi: 10.1038/s41467-023-40523-3 (PMC10480438; doi:10.1038/s41467-023-40523-3)
Supplement: Supplementary file 1 — Supplementary Information [file 41467_2023_40523_MOESM1_ESM.pdf]

Supplementary Information

Fine-scale collective movements reveal present, past and future dynamics of a multilevel society in Przewalski's horses

Katalin Ozogány[1,2*], Viola Kerekes[3], Attila Fülöp[1,2,4,5,6], Zoltán Barta[1,2†], Máté Nagy[7,8,9,10†*]

[1] ELKH-DE Behavioural Ecology Research Group, University of Debrecen, Egyetem tér 1, H-4032 Debrecen, Hungary

[2] Department of Evolutionary Zoology and Human Biology, University of Debrecen, Egyetem tér 1, H-4032 Debrecen, Hungary

[3] Hortobágy National Park Directorate, Sumen u. 2, H-4024 Debrecen, Hungary

[4] Evolutionary Ecology Group, Hungarian Department of Biology and Ecology, Babeș-Bolyai University, str. Clinicilor 5-7, RO-400006 Cluj-Napoca, Romania

[5] Centre for Systems Biology, Biodiversity and Bioresources (3B), Babeș-Bolyai University, str. Clinicilor 5-7, RO-400006 Cluj-Napoca, Romania

[6] STAR-UBB Institute of Advanced Studies in Science and Technology, Babeș-Bolyai University, str. Mihail Kogălniceanu 1, RO-400084 Cluj-Napoca, Romania

[7] MTA-ELTE "Lendület" Collective Behaviour Research Group, Hungarian Academy of Sciences, Pázmány P. stny. 1A, H-1117 Budapest, Hungary

[8] Department of Biological Physics, Eötvös Loránd University, Pázmány P. stny. 1A, H-1117 Budapest, Hungary

[9] MTA-ELTE Statistical and Biological Physics Research Group, Hungarian Academy of Sciences, Pázmány P. stny. 1A, H-1117 Budapest, Hungary

[10] Department of Collective Behaviour, Max Planck Institute of Animal Behavior, Universitätsstraße 10, Konstanz 78457, Germany

† These authors contributed equally to this work.

* Corresponding authors: katalin.ozogany@gmail.com, nagymate@hal.elte.hu

Supplementary Note 1: Movement variables and the social structure

The two movement variables studied, d and C, were not independent from each other, as the movement similarity of a horse pair, unsurprisingly, decreased with the pair's distance (e.g. in the first recording session Pearson's $r = -0.594$, $p < 0.0001$, $n = 230$ for adult female pairs from same harems, and Pearson's $r = -0.327$, $p < 0.0001$, $n = 6211$ for adult female pairs from different harems, randomisation tests; Supplementary Figure 2a).

The distribution of pairwise distances, d, changed across the studied time-scales: (i) the distribution of distances calculated in a single moment d_0 (measured in a still frame from the video), has only one peak, thus the multilevel structure of the herd is not detectable based on a still image, (ii) when calculating distance over a 5-min recording session d_{5min}, the distribution of distances shows two peaks, thus multilevel structure is detectable, (iii) in the distribution of distances averaged over five observation sessions from different days, $\bar{d}$, the two peaks are well separated, the multilevel structure is clear (Supplementary Figure 2c-e). Furthermore, in the distribution of $\bar{d}$ the minimum point, that is around 20 m, roughly divides the same-harem and different-harem pairs (Figure 4a). Also, based on the distribution of $\bar{d}$ the typical pairwise distances are 5-10 m within harems, while the longest size of the herd (length in the direction of movement) is around 150 m (Supplementary Figure 2e).

To test whether harem memberships can be determined based solely on movement variables, we performed a cluster analysis and calculated the accuracy of classifying individuals into harems. We performed agglomerative hierarchical clustering with Ward's method on the pairwise distances averaged over a single observation session, as dissimilarity measure, and classified individuals in a given number of clusters ($n = 43$, as there were 31 known harem groups and 12 bachelor groups in the population at the time of the study). In the five observation sessions, clustering resulted mainly in two types of groups: groups of harem-living individuals and single-sex bachelor groups; mixed groups were found with a very low rate (12%). The ratio of correctly classified harem-living individuals, (i) when an individual is classified into the same cluster as its harem stallion, was 76%; (ii) when an individual is classified into a cluster where the majority of cluster members are from the individual's harem, was 90%. Note that based only on clustering bachelor groups cannot be distinguished from harem groups. We found that pairwise distances are more suitable in classifying individuals into sub-units than movement similarity.

Although two social levels were clearly indicated by the two-peaked distribution of pairwise distances, the multilevel structure is less clear considering the movement similarities, as the distribution of C has only one peak, both for a single 5-min session and for averaged sessions (Supplementary Figure 2f-g). Further investigation, however, shows that the distribution of $\bar{C}$ changes abruptly when considering different $\bar{d}$ intervals (Supplementary Figure 8b). According to the shape of the distribution of $\bar{C}$, three main ranges of distances can be distinguished, namely, (i) a distance range below cca. 20 m,

where most of the pairs have very similar movement ($\bar{C}$ > 0.9), and this is the range where pairs with the highest movement similarity ($\bar{C}$ > 0.95) are found almost exclusively, (ii) a distance range between cca. 20-40 m, where pairs move with a moderate but still relatively high similarity ($0.85 < \bar{C} < 0.95$), and (iii) a distance range over cca. 40 m, where pairs with lower movement similarity ($\bar{C}$ < 0.8) also appear (Supplementary Figure 2b, Supplementary Figure 8b-c). The first distance range can be interpreted as the range of individual pairs from the same harem, the second one as the range of individual pairs from neighbouring harems (with relatively aligned movement), and the third one as the range of individual pairs from further, i.e. not neighbouring, harems (with less aligned movement). Note that 20 m is roughly a harem radius, thus 40 m is the second-neighbour harem distance. Inter-harem individual pairs are not randomly distributed in the second and third ranges, which suggests that, when considering longer time scales, some of the harems are generally closer to each other, while others prefer to stay further from the others. Thus, the movement variables averaged over observation sessions can be used to reveal the strength of association between the harems.

Supplementary Note 2: Results on physical distances

In our analyses we constructed a social network based on proximity and studied distances of individuals and harems in the social network (network distance, i.e. length of the shortest path between nodes). Thus, relationships found on network distances mean that similar results can be shown in physical distances as well. Although, if an effect is not significant in network distances does not necessarily mean that it is not significant in physical distances because of the data loss during the network construction (e.g. when cutting bonds at a given threshold, see Methods). For the cases not significant in network distances, we provide results in physical distances.

Effect of female kinship within harems

Close kin adult females from the same harem were not significantly closer to each other on average than more distant relatives, when comparing full or half-siblings ($p = 0.084$, $n_1 = 15$, $n_2 = 205$) and parent-offspring pairs to more distantly related pairs ($p = 0.370$, $n_1 = 10$, $n_2 = 205$, randomisation tests).

Effect of female kinship between harems

Distance between harems containing parent-offspring adult female pairs did not differ significantly from harems not containing close kin females ($p = 0.359$, $n_1 = 14$, $n_2 = 206$, randomisation test; excluding harems with familiar females).

Effect of past associations of stallions

Common past membership of stallions did not seem to affect proximity of their harems, as harems of non-related stallions were not closer if the stallions lived longer in the same parental harem in the past (Pearson's r = -0.044, p = 0.210, n = 381, randomisation test).

Supplementary Note 3: Relationships between harem traits

Harem traits assessed in the study were not independent from each other. Older harems belonged to more experienced (Pearson's r = 0.724, p < 0.0001, n = 31) and older males (Pearson's r = 0.659, p < 0.001, n = 30), while the average age of adult female members also increased with the harem's age (Pearson's r = 0.569, p < 0.001, n = 31, randomisation tests). The harem size (counting the adult and subadult members as well) increased with the harem's age (Pearson's r = 0.503 p = 0.003, n = 31), as well as the number of subadult offspring in the harem (Pearson's r = 0.555, p = 0.002, n = 31), while the number of adult females did not increase significantly with the harem's age (Pearson's r = 0.268, p = 0.0748, n = 31, randomisation tests; Supplementary Figure 7).

Supplementary Note 4: Robustness of results on proximity networks

To show robustness of results obtained on the d_{th} = 53 m network, we assessed the correlation of closeness centrality of harems in the d_{th} = 47.7 m (10% lower threshold) and d_{th} = 58.3 m (10% higher threshold) network with harem traits. The relationship between closeness centrality and harem traits was not sensitive to the value of d_{th} distance threshold, since a 10% lower and a 10% higher threshold produced similar results as seen for the d_{th} = 53 m network. Closeness centrality in the d_{th} = 47.7 m network was positively correlated with harem's age (Pearson's r = 0.512, p < 0.001, n = 31), stallion's experience (Pearson's r = 0.463, p = 0.003, n = 31), stallion's age (Pearson's r = 0.460, p = 0.006, n = 30), and harem size (Pearson's r = 0.479, p = 0.002, n = 31, randomisation tests). Similarly, for d_{th} = 58.3 m network, closeness centrality was positively correlated with harem's age (Pearson's r = 0.553, p < 0.001, n = 31), stallion's experience (Pearson's r = 0.604, p < 0.001, n = 31), stallion's age (Pearson's r = 0.595, p < 0.001, n = 30), and harem size (Pearson's r = 0.406, p = 0.013, n = 31, randomisation tests).

The results were not sensitive for different centrality measures, as we obtained similar correlations when using closeness and degree centrality in comparisons. Degree centrality in the d_{th} = 53 m network was positively correlated with harem's age (Pearson's r = 0.604, p < 0.001, n = 31), stallion's experience (Pearson's r = 0.716, p < 0.0001, n = 31), stallion's age (Pearson's r = 0.652, p = 0.0001, n = 30), and harem size (Pearson's r = 0.472, p = 0.006, n = 31, randomisation tests; Supplementary Figure 6).

Supplementary Figures

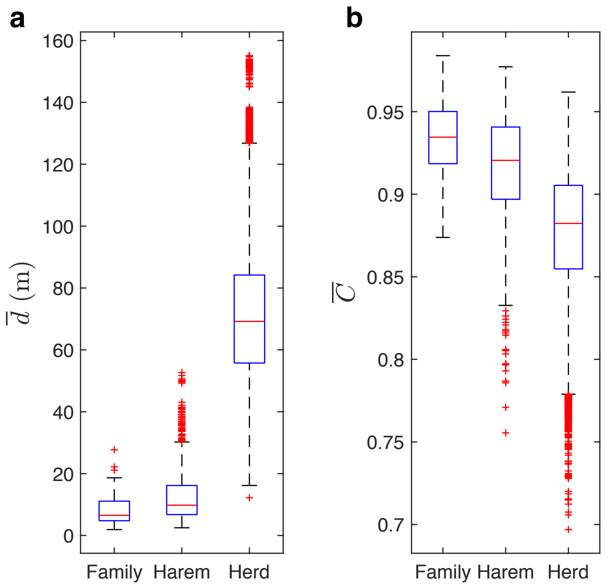

Supplementary Figure 1. Pairwise distance and movement similarity between horse pairs.

a Distance and **b** movement similarity of horse pairs averaged over five 5-minute observation sessions belonging to the (i) same family, "Family" (n =70), (ii) different family but same harem, "Harem" (n = 711), and (iii) different harem but same herd, "Herd" (n = 21374). The pairs' distance increases while movement similarity decreases from "Family" towards "Herd". Boxes range from the 25th to 75th percentile, while central marks denote medians, whiskers extend to extreme data points not considered outliers (marked in red).

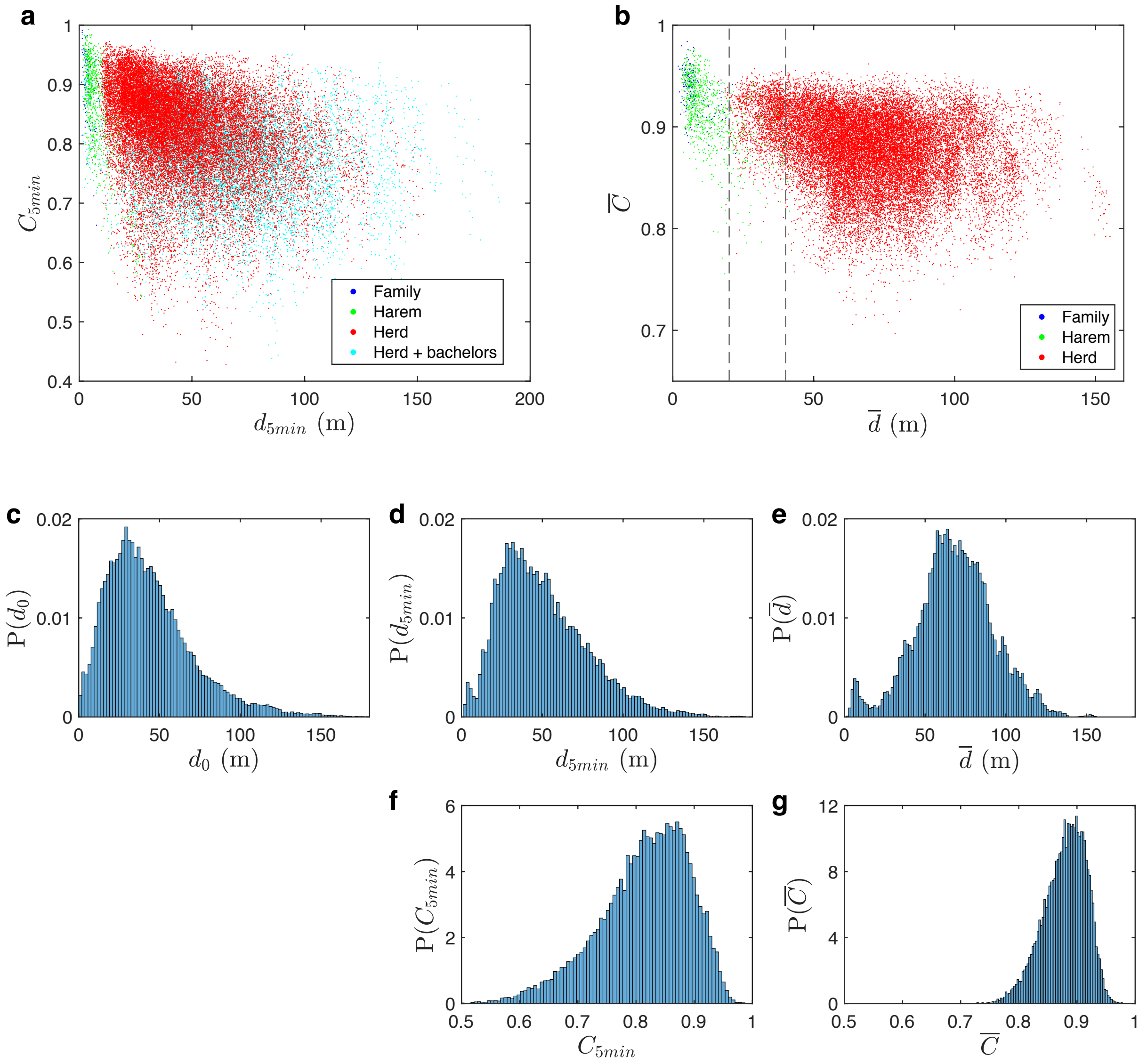

Supplementary Figure 2. Local pair-interactions: pairwise distances and movement similarities between individuals, during collective movements. a Movement similarity, *C*, against pairwise distance, *d*, between horse pairs in a 5-min movement observation session, and **b** averaged over five 5-min observation sessions from different days. Colours denote pairs from (i) the same family, "Family" (blue), (ii) different family but same harem, "Harem" (green), (iii) different harem but same herd, "Herd" (red); and (iv) a bachelor and harem-living individual pair, "Herd + bachelors" (cyan). Vertical dashed lines show roughly the thresholds, where the distribution of *C* changes (see also Supplementary Figure 8c and Supplementary Note 1). **c-e** Distribution of pairwise distances over different time scales: distances measured in a single moment (still image) (c), averaged over a 5-min observation session (d), averaged over five 5-min observation sessions (e). Position of the first peak (d, e) shows typical distance between harem members, while the position of the minimum point between the two peaks (d, e) shows the typical half-distance between the nearest neighbouring harems. The two peaks, which support the existence of the two levels in the society, cannot be seen in distances measured at a moment (c), but

become obvious after several minutes of observation (d, e). **f-g** Distribution of movement similarity over different time scales: during a 5-min observation session (f) and averaged over five 5-min observation sessions (g). Note, as movement similarity is a dynamic measure it cannot be calculated for a single moment.

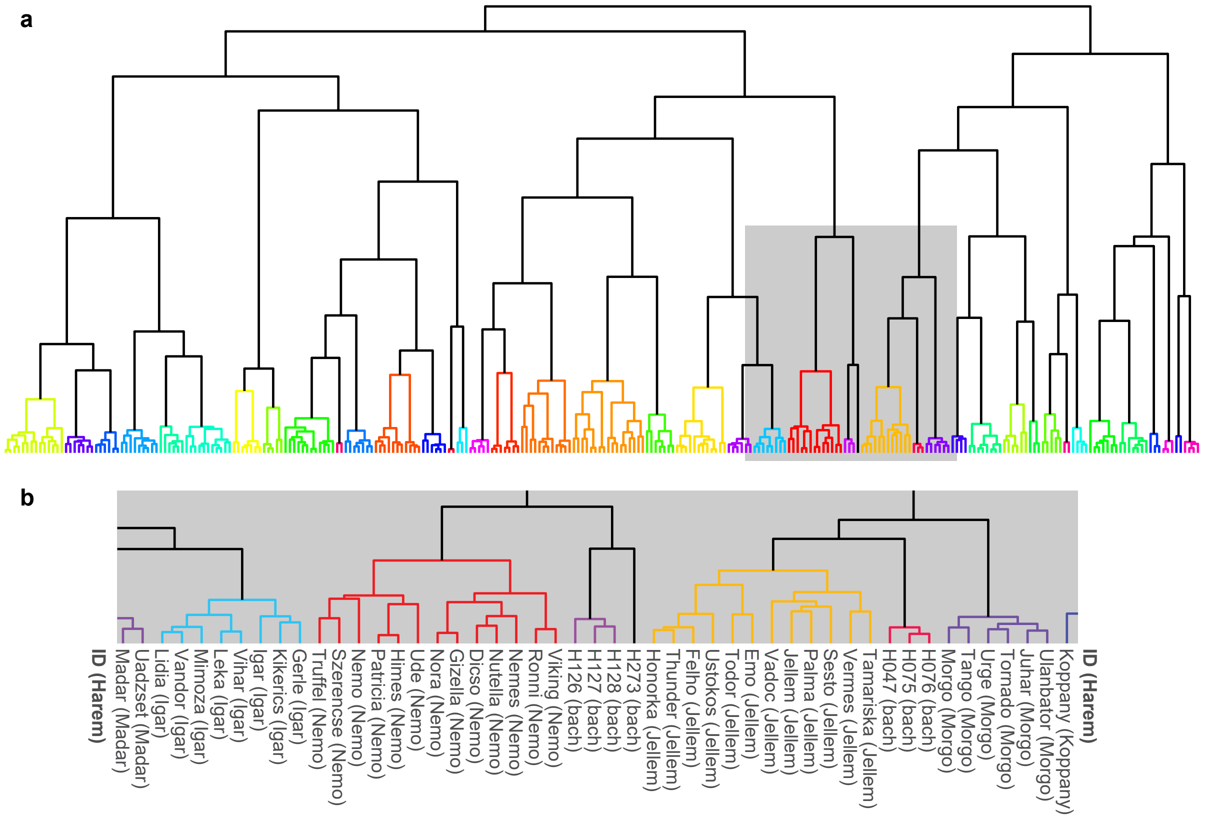

Supplementary Figure 3. Hierarchical cluster analysis (HCA) of distances in a 5-min movement observation session. a Dendrogram of the agglomerative HCA using the pairwise distances between individuals as dissimilarity measure. Colours denote the identified clusters of individuals (for a given number of clusters n = 43, which is the number of harem and bachelor groups based on the national park's records). **b** Detailed view of a part of the dendrogram, where individual IDs (names) are indicated, as well as the harems the individuals actually belong to based on the national park's records in brackets (or "bach" for bachelor males).

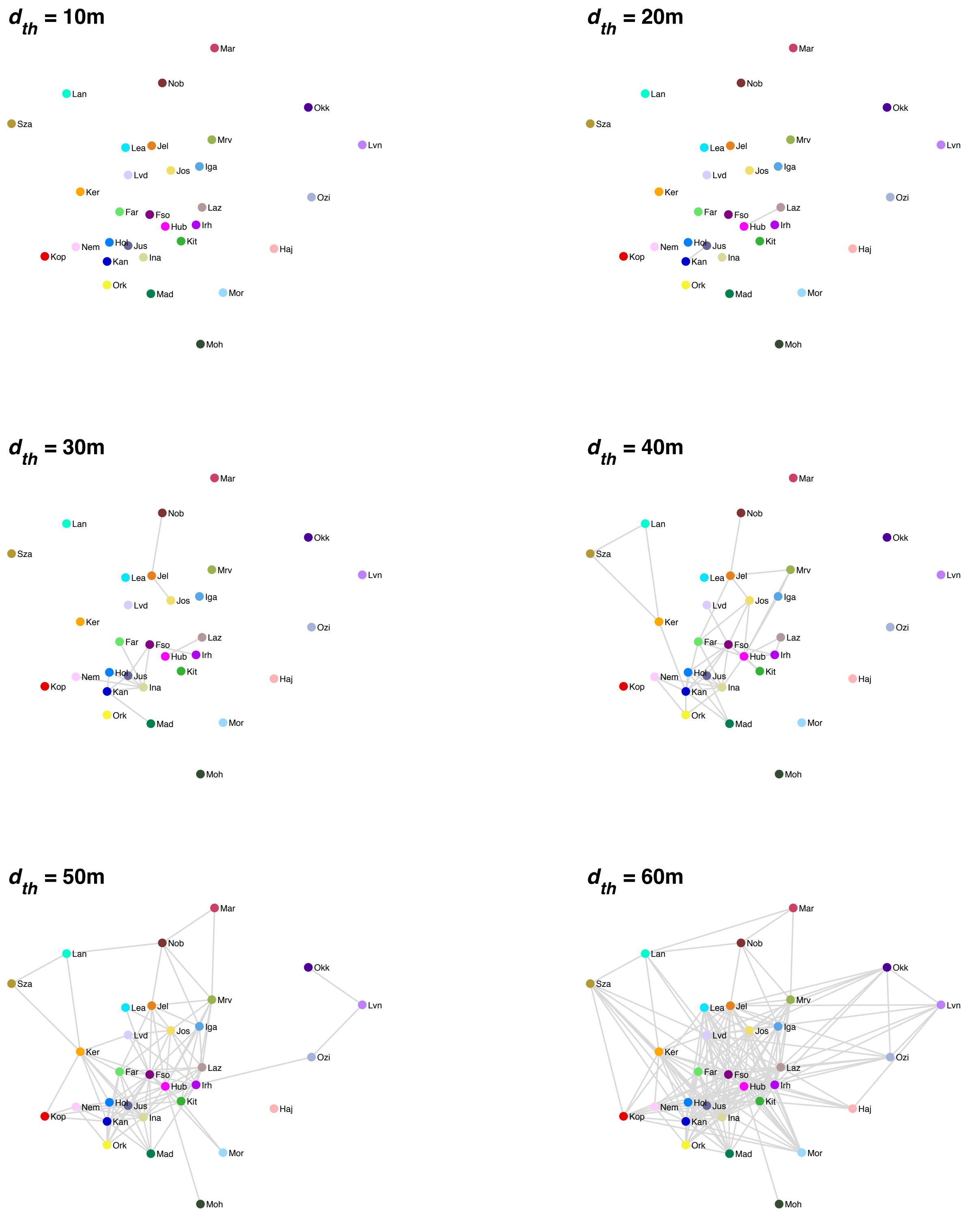

Supplementary Figure 4. Network of harems based on individual pairwise distances, with various d_{th} thresholds. Nodes correspond to harems in the population (colour coding matches Figure 4d), three letter labels indicate harem IDs (name of the harem stallion). An edge is drawn between two harems if any of their members are closer on average than a given threshold, $\bar{d} < d_{th}$.

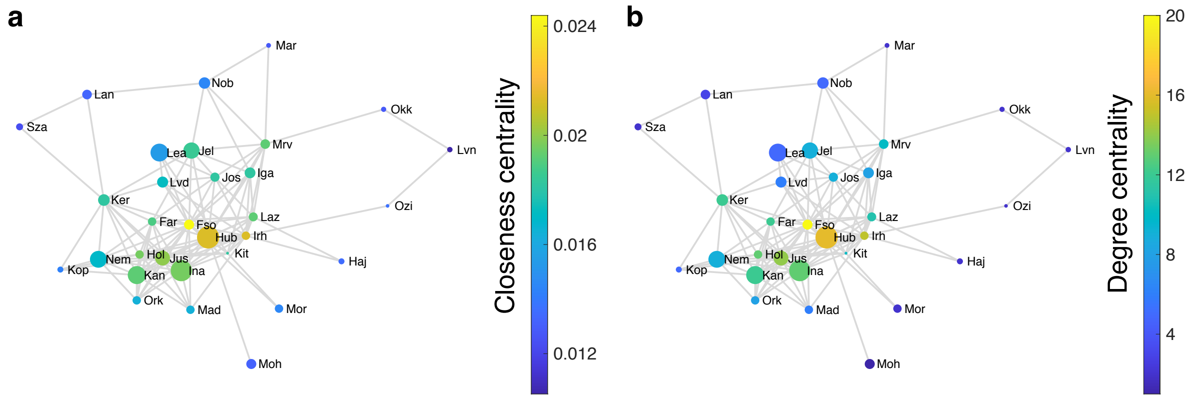

Supplementary Figure 5. Centrality of harems in the d_{th} = 53 m network. a Closeness centrality, i.e. the reciprocal of the mean shortest path distance from all other reachable nodes. **b** Degree centrality, i.e. the number of links a node has. Nodes correspond to harems, three letter labels indicate harem IDs (name of the harem stallion), node size is proportional to harem size, while node colour indicates the harem's network centrality according to the scale to the right.

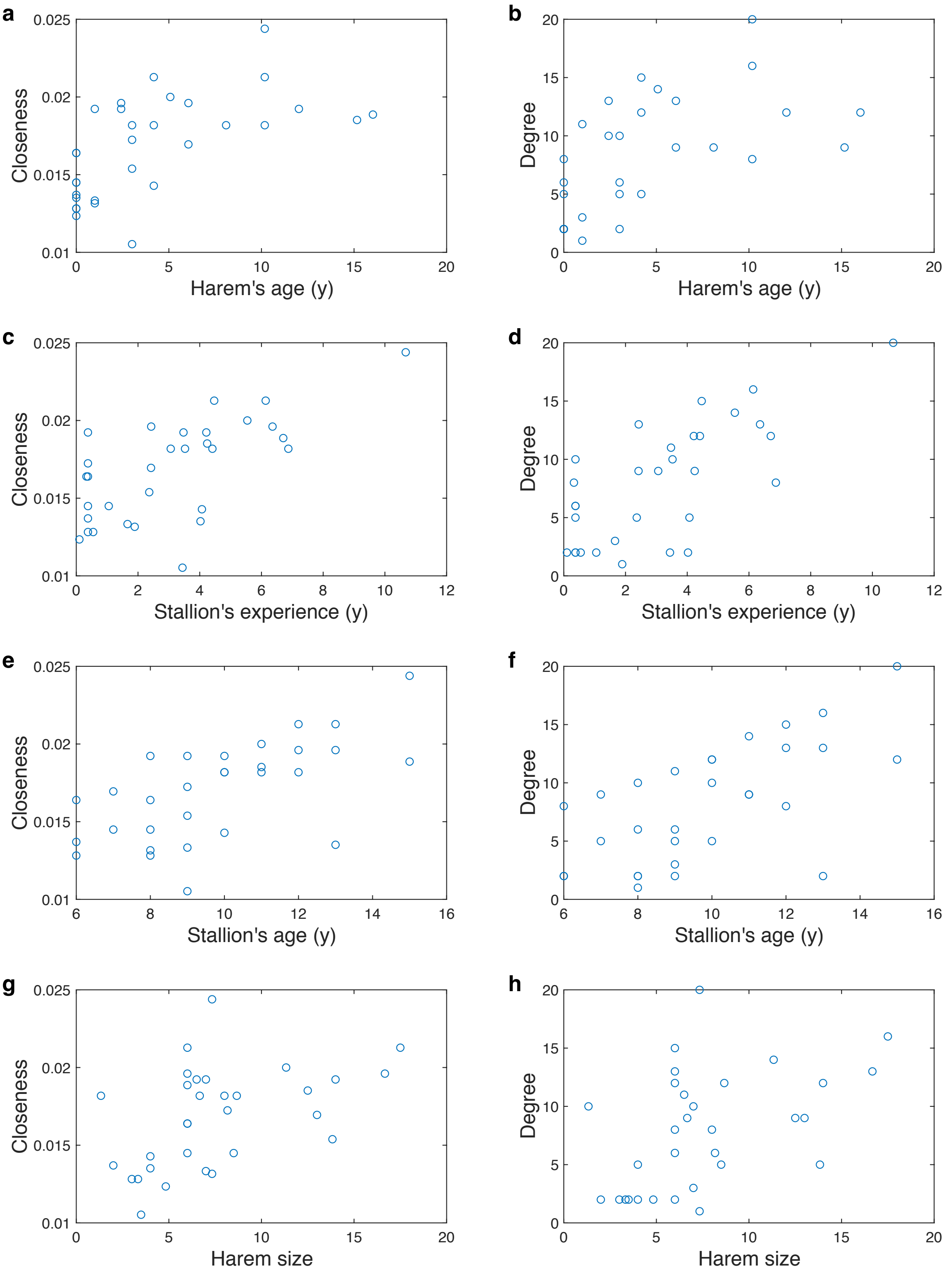

Supplementary Figure 6. Robustness of results for different centrality measures. Closeness (a, c, e, g) and degree centrality (b, d, f, h) of harems in the d_{th} = 53 m network against harem traits. The relationship between network centrality and harem traits was not sensitive to the centrality measure we used, as we obtained similar results when using closeness and degree centrality in comparisons. Centrality of harems was positively correlated with harem's age (a, b), stallion's experience in harem keeping (c, d), stallion's age (e, f), and harem size (g, h; see Supplementary Note 4 for details).

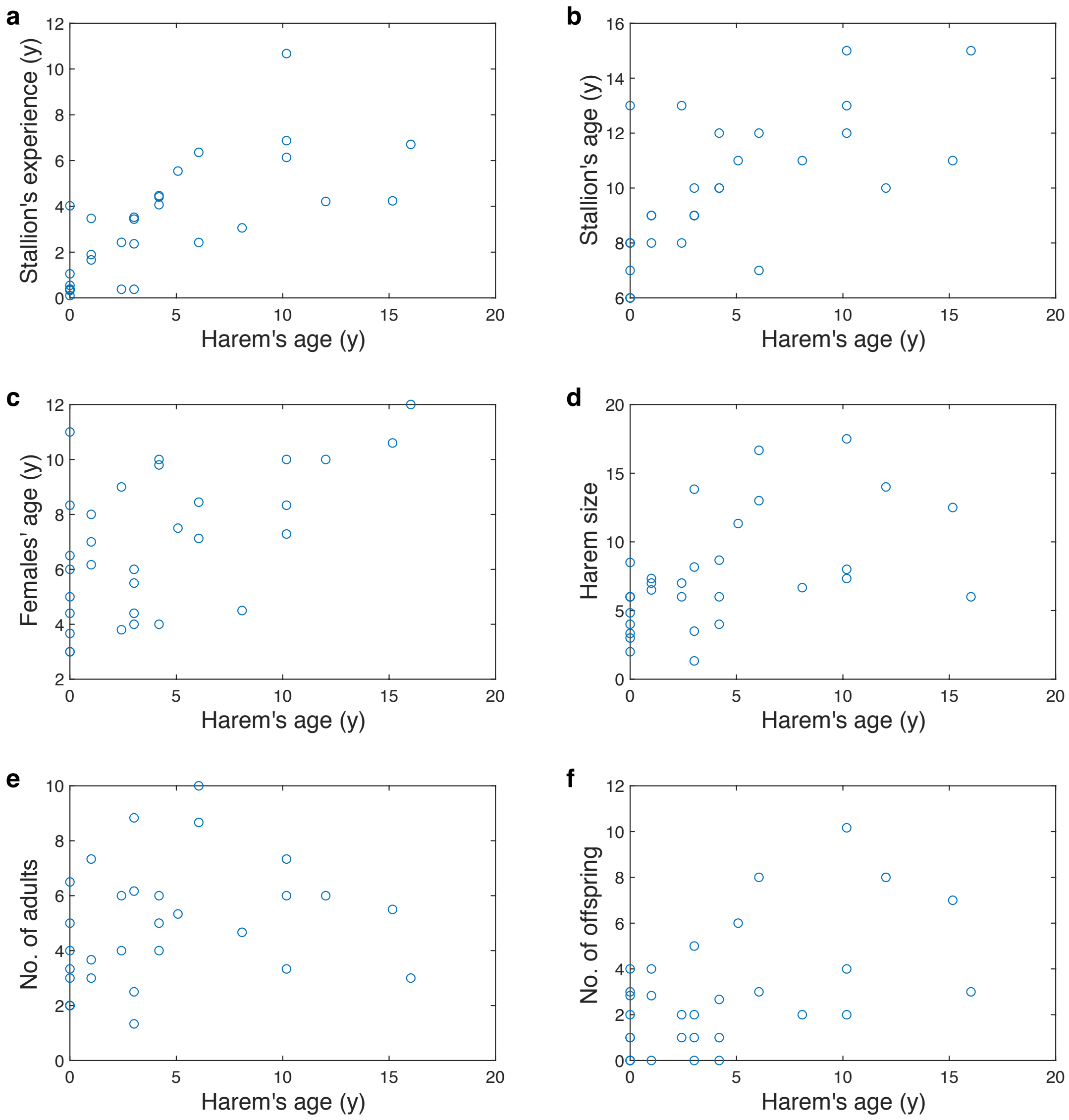

Supplementary Figure 7. Relationships between harem traits. Different harem traits against the harem's age in years. Older harems belonged to more experienced (a) and older males (b), while the average age of adult female members also increased with the harem's age (c). The harem size (counting the adult and subadult members as well) increased with the harem's age (d), as well as the number of subadult offspring in the harem (f), while the number of adult females did not increase significantly with the harem's age (e; see Supplementary Note 3 for details).

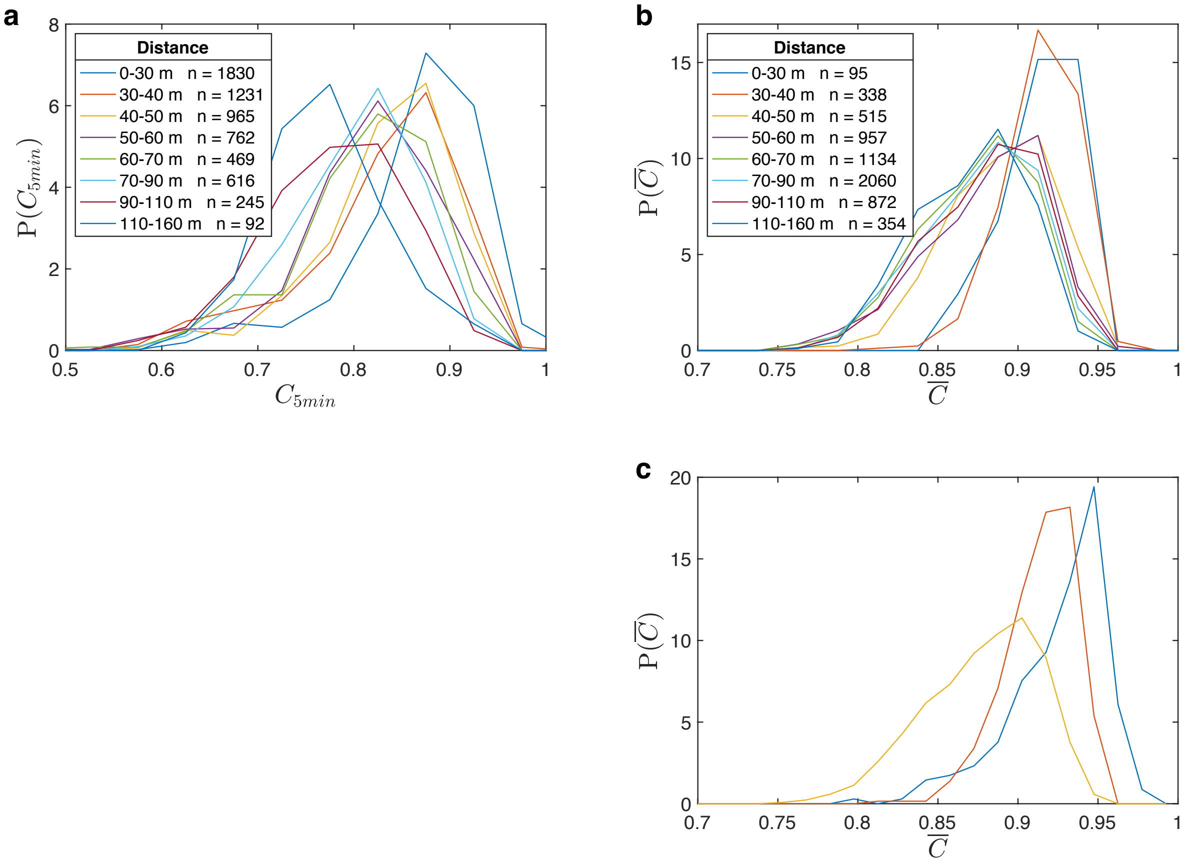

Supplementary Figure 8. **Distribution of movement similarity for different distance ranges.**

a Distribution of movement similarity in a 5-min observation session, and **b** for averaged sessions, for adult female pairs belonging to different harems and for different distance ranges between the individuals. For averaged sessions the distribution of $\bar{C}$ changes abruptly at d = 40 m. Notably, 40 m is roughly the distance between the nearest neighbour harems. **c** Summarized distributions of movement similarity for the three different ranges: (i) 0-20 m distance range of same-harem female pairs (blue), (ii) 20-40 m distance range of different-harem female pairs (red), (iii) >40 m distance range of different-harem female pairs (orange). As inter-harem pairs are not randomly distributed in the second and third ranges, it suggests that some harems stay closer together, while others prefer to stay further from the others.

Supplementary Tables

Supplementary Table 1. Number of individuals involved in the study. The number of individuals tracked on the aerial videos, from which the number of individually identified and unidentified horses versus the population size (including horses that are members of a harem and bachelor males), on the five observation days, respectively. Bachelor males were not identified on the videos, while two individuals from a harem could not be identified in two sessions (17 Aug 2018 and 2 Oct 2018). In two sessions (13 Sep 2018 and 2 Oct 2018) not all individuals appeared in the videos.

Observation session (date)	Tracked individuals	Identified individuals	Unidentified individuals	Population size	Harem members	Bachelor males
17 Aug 2018	278	236	42	278	238	40
24 Aug 2018	278	238	40	278	238	40
5 Sep 2018	278	238	40	278	238	40
13 Sep 2018	249	218	31	278	238	40
2 Oct 2018	267	231	36	273	233	40

Supplementary Table 2. Summary of all statistical tests and corrections for multiple comparisons. Here, we report all statistical tests (one-sided randomisation tests) performed with sample sizes (n), significance levels (p) and corrected significance levels (α_1) using the Šidák correction[1]. Significance levels in bold indicate tests where $p < \alpha_1$. We note here that this is a conservative method derived by assuming that the individual tests are independent. This is not true in several cases in our study, thus, using the corrected values for significance comes at the cost of increasing the probability of producing false negatives and reducing statistical power. So, we present α_1 Šidák corrected significance levels as a guideline. The statistical tests are reported in the order of appearance in the paper.

**Comparison**	**Order**	**Test name**	***n***	***p***	α_1
distance, harem vs. herd, 1st session	1	randomisation	707; 20959	**10^{-7}**	0.050
movement similarity, harem vs. herd, 1st session	2	randomisation	707; 20959	**10^{-7}**	0.025
distance, harem vs. herd, averaged data	3	randomisation	711; 21374	**10^{-7}**	0.017
movement similarity, harem vs. herd, averaged data	4	randomisation	711; 21374	**10^{-7}**	0.013
distance, family vs. harem, 1st session	5	randomisation	70; 707	**10^{-7}**	0.010
movement similarity, family vs. harem, 1st session	6	randomisation	70; 707	**4×10^{-5}**	0.009
distance, family vs. harem, averaged data	7	randomisation	70; 711	**0.0003**	0.007
movement similarity, family vs. harem, averaged data	8	randomisation	70; 711	**3×10^{-6}**	0.006
distance, herd vs. herd + bachelors, 1st session	9	randomisation	20959; 8778	**10^{-7}**	0.006
movement similarity, herd vs. herd + bachelors, 1st session	10	randomisation	20959; 8778	**2×10^{-7}**	0.005
network distance, sibling vs. more distantly related, same harem adult female pairs	11	randomisation	15; 199	0.079	0.005
network distance, parent-offspring vs. more distantly related, same harem adult female pairs	12	randomisation	9; 199	0.334	0.004
ratio of sibling adult females, inside harems vs. population average	13	randomisation	31	0.107	0.004
correlation, network distance vs. t_{past}, same harem non-related adult female pairs	14	randomisation	199	0.006	0.004
network distance, harems of sibling (full and half together) vs. more distantly related stallions	15	randomisation	53; 411	**0.0004**	0.003
network distance, harems of full sibling vs. half-sibling stallions	16	randomisation	9; 44	0.046	0.003
correlation, network distance vs. t_{past*}, harems of non-related stallions	17	randomisation	381	0.26	0.003
network distance, harems with sibling vs. more distantly related females, $t_{past\_af\_harem} = 0$	18	randomisation	65; 206	0.004	0.003
network distance, harems with mother-daughter vs. more distantly related females, $t_{past\_af\_harem} = 0$	19	randomisation	14; 206	0.342	0.003
network distance, harems that have exchanged adult females in the previous 2 years vs. that have not	20	randomisation	27; 438	0.021	0.003
network distance, harems that have exchanged adult females in the subsequent 2 years vs. that have not	21	randomisation	24; 441	0.017	0.002
correlation, network distance vs. t_{future}, non-related same harem adult female pairs	22	randomisation	199	0.012	0.002
movement similarity, non-related adult females from different harems with $t_{future} > 90$ vs. $t_{future} = 0$ females in their harems, $t_{past} = 0$	23	randomisation	109	0.007	0.002
correlation, harem's closeness centrality vs. harem's age	24	randomisation	31	**0.0003**	0.002

correlation, harem's closeness centrality vs. stallion's experience	25	randomisation	31	**2×10^{-5}**	0.002
correlation, harem's age vs. stallion's experience	26	randomisation	31	**8×10^{-7}**	0.002
correlation, harem's closeness centrality vs. stallion's age	27	randomisation	30	**0.0002**	0.002
correlation, stallion's experience vs. stallion's age	28	randomisation	30	**10^{-7}**	0.002
correlation, harem's closeness centrality vs. harem size	29	randomisation	31	0.005	0.002
correlation, harem's closeness centrality vs. adult harem size	30	randomisation	31	0.061	0.002
correlation, average distance from the closest bachelor male vs. harem's closeness centrality	31	randomisation	31	0.239	0.002
correlation, average distance from the closest bachelor male vs. no. of adult females in harem	32	randomisation	31	0.010	0.002
correlation, distance vs. movement similarity, same harem adult female pairs, 1st session	33	randomisation	230	**8×10^{-8}**	0.002
correlation, distance vs. movement similarity, different harem adult female pairs, 1st session	34	randomisation	6211	**10^{-7}**	0.002
distance, sibling vs. more distantly related, same harem adult female pairs	35	randomisation	15; 205	0.084	0.001
distance, parent-offspring vs. more distantly related, same harem adult female pairs	36	randomisation	10; 205	0.370	0.001
distance, harems with mother-daughter vs. more distantly related females, $t_{past\_af\_harem} = 0$	37	randomisation	14; 206	0.359	0.001
correlation, distance vs. t_{past*}, harems of non-related stallions	38	randomisation	381	0.210	0.001
correlation, harem's degree centrality vs. harem's age	39	randomisation	31	**0.0003**	0.001
correlation, harem's degree centrality vs. stallion's experience	40	randomisation	31	**6×10^{-6}**	0.001
correlation, harem's degree centrality vs. stallion's age	41	randomisation	30	**0.0001**	0.001
correlation, harem's degree centrality vs. harem size	42	randomisation	31	0.006	0.001

Supplementary references

1. Sidak, Z. Rectangular Confidence Regions for the Means of Multivariate Normal Distributions. *J. Am. Stat. Assoc*. **62**, 626–633 (1967).
