# Supplementary material for: Fine-scale collective movements reveal present, past and future dynamics of a multilevel society in Przewalski’s horses
Source: Nat Commun. 2023 Sep 5;14:5096. doi: 10.1038/s41467-023-40523-3 (PMC10480438; doi:10.1038/s41467-023-40523-3)
Supplement: Supplementary file 3 — Description of additional supplementary files [file 41467_2023_40523_MOESM3_ESM.pdf]

# Description of Additional Supplementary Files Document

**Supplementary Movie 1**

Sample aerial video of the collective movement of Przewalski's horses at Pentezug reserve, Hortobágy National Park, Hungary. The video was taken by a higher drone to provide a large-scale top view for tracking individuals and the background to get coordinates and movement in an earth-fixed coordinate system. A drone flying at lower altitude was used to capture footage of the horses for a detailed view allowing individual recognition.

**Supplementary Movie 2**

Sample clip showing the position of all the horses in the herd from tracked drone footage. Positions (x, y in meters) are shown in an earth-fixed coordinate system. Markers represent identified individuals, colour-coded based on the group they belong to (out of 31 harems), or shown as grey "x" in the case of bachelor males (i.e. males that are not part of a harem).
