# Supplementary material for: Fine-scale collective movements reveal present, past and future dynamics of a multilevel society in Przewalski’s horses
Source: Nat Commun. 2023 Sep 5;14:5096. doi: 10.1038/s41467-023-40523-3 (PMC10480438; doi:10.1038/s41467-023-40523-3)
Supplement: Supplementary file 6 — Reporting Summary [file 41467_2023_40523_MOESM6_ESM.pdf]

nature portfolio

Corresponding author(s): Máté Nagy, Katalin Ozogány

Last updated by author(s): Jul 26, 2023

# Reporting Summary

Nature Portfolio wishes to improve the reproducibility of the work that we publish. This form provides structure for consistency and transparency in reporting. For further information on Nature Portfolio policies, see our Editorial Policies and the Editorial Policy Checklist.

## Statistics

For all statistical analyses, confirm that the following items are present in the figure legend, table legend, main text, or Methods section.

| n/a | Confirmed | |
|---|---|---|
| ☐ | ☒ | The exact sample size (*n*) for each experimental group/condition, given as a discrete number and unit of measurement |
| ☐ | ☒ | A statement on whether measurements were taken from distinct samples or whether the same sample was measured repeatedly |
| ☐ | ☒ | The statistical test(s) used AND whether they are one- or two-sided<br>*Only common tests should be described solely by name; describe more complex techniques in the Methods section.* |
| ☒ | ☐ | A description of all covariates tested |
| ☐ | ☒ | A description of any assumptions or corrections, such as tests of normality and adjustment for multiple comparisons |
| ☐ | ☒ | A full description of the statistical parameters including central tendency (e.g. means) or other basic estimates (e.g. regression coefficient) AND variation (e.g. standard deviation) or associated estimates of uncertainty (e.g. confidence intervals) |
| ☐ | ☒ | For null hypothesis testing, the test statistic (e.g. *F*, *t*, *r*) with confidence intervals, effect sizes, degrees of freedom and *P* value noted<br>*Give P values as exact values whenever suitable.* |
| ☒ | ☐ | For Bayesian analysis, information on the choice of priors and Markov chain Monte Carlo settings |
| ☒ | ☐ | For hierarchical and complex designs, identification of the appropriate level for tests and full reporting of outcomes |
| ☐ | ☒ | Estimates of effect sizes (e.g. Cohen's *d*, Pearson's *r*), indicating how they were calculated |

*Our web collection on statistics for biologists contains articles on many of the points above.*

## Software and code

Policy information about availability of computer code

| | |
|---|---|
| Data collection | Blender 2.79b, Python 2.7, Microsoft Excel for Mac 16.54 |
| Data analysis | Custom-written codes in MATLAB R2021a and CUDA 11.5, provided in GitHub repository at https://github.com/katalinozogany/wildhorse_mls. |

For manuscripts utilizing custom algorithms or software that are central to the research but not yet described in published literature, software must be made available to editors and reviewers. We strongly encourage code deposition in a community repository (e.g. GitHub). See the Nature Portfolio guidelines for submitting code & software for further information.

## Data

Policy information about availability of data

All manuscripts must include a data availability statement. This statement should provide the following information, where applicable:

- Accession codes, unique identifiers, or web links for publicly available datasets
- A description of any restrictions on data availability
- For clinical datasets or third party data, please ensure that the statement adheres to our policy

Data generated during the analyses that support the findings of this study have been deposited on Github at https://github.com/katalinozogany/wildhorse_mls. The raw data are available under restricted access for nature conservation reasons, access can be obtained from the Hortobágy National Park Directorate and the first author on reasonable request.

## Human research participants

Policy information about studies involving human research participants and Sex and Gender in Research.

| | |
|---|---|
| Reporting on sex and gender | The study did not involve human participants. |
| Population characteristics | The study did not involve human participants. |
| Recruitment | The study did not involve human participants. |
| Ethics oversight | The study did not involve human participants. |

Note that full information on the approval of the study protocol must also be provided in the manuscript.

# Field-specific reporting

Please select the one below that is the best fit for your research. If you are not sure, read the appropriate sections before making your selection.

☐ Life sciences ☐ Behavioural & social sciences ☒ Ecological, evolutionary & environmental sciences

For a reference copy of the document with all sections, see nature.com/documents/nr-reporting-summary-flat.pdf

# Ecological, evolutionary & environmental sciences study design

All studies must disclose on these points even when the disclosure is negative.

| | |
|---|---|
| Study description | Long-term population monitoring, genetic sampling, short-term high-resolution aerial tracking of the development and details of 1 population over 22 years, containing maximum 328 horses and 31 harems. |
| Research sample | We studied the collective movements of Przewalski's horses (Equus ferus przewalskii) in Hortobagy National Park (HNP), located in eastern Hungary (47°31'3.3"N 21°5'34.1"E). In 1997 Przewalski's horses were introduced to the "Pentezug" Reserve, a 3000 ha steppe area within HNP, as part of an ecological habitat management scheme by large grazers: wild horses and cattle. The conditions are close to wild, human activities are restricted, and the horses are not fed or watered. The total number of large grazers was around 680 during our observation period (273-278 Przewalski's horses and cca. 400 Heck cattle). The Przewalski's horse population originated from 31 founder individuals which arrived in the reserve between 1997-2017 from different European zoological gardens, though during the aerial observations only two founder individuals were still alive. |
| Sampling strategy | All individuals except young males while bachelors are individually identified on the basis of natural pelage colouring and characteristic features and injuries, supported by a photo catalogue, harem composition lists and DNA-fingerprint database. Data collection includes records on life history events, like birth date, death date, identity of parents and changes in harem membership. Biopsy samples are taken from each individual at the age of 1 year, which serves for determining parentage and updating the DNA-fingerprint database. Parentage records based on observations are supported by DNA-tests in most cases (74%). Males are individually known until leaving the parental harem, then again when they acquire a harem or when they die, but membership information is lacking while in bachelor groups, which have a less consistent membership. Harem composition lists are updated with a temporal resolution of 12 +/- 8 observations per year. Individual traits known from population monitoring include age, sex, role in the social system (adult female, subadult individual in parental harem, harem stallion, bachelor), relatedness to other individuals, and current and past records on harem membership. |
| Data collection | We collected data by performing aerial video recordings of the herd on the move with two DJI Phantom 4 drones simultaneously at 4k resolution and 25fps (frames-per-second). Prior to the study we tested the disturbance of drones with decreasing flying altitudes and observed that horses started to avoid drones at around 3-4m flying altitudes. A drone flying high (100-300m from the ground) recorded the moving herd and provided the image for movement tracking, while a drone flying low (10-30m from the ground) scanned through the whole herd and provided a detailed image for individual identification. The recordings of the two drones were synchronised, and thus identities of individuals could be matched with the tracked trajectories. The top view drone's video was processed with the Motion Tracking function of Blender v2.79b45 and the spatial position of each individual was determined in each frame, with 12.5 frame/sec temporal resolution. Aerial videos were recorded by Katalin Ozogány and Tímea Szabados (Hortobágy National Park Directorate). |
| Timing and spatial scale | Population monitoring was carried out between 1997 and 2020 reported in this paper. The aerial observation contains five sessions, recorded on five different days, 1-2 weeks apart (August 17, August 24, September 5, September 13, and October 2 in 2018), during daylight hours (between 8am – 3pm). |
| Data exclusions | Bachelor males where excluded from parts of the analysis due that their identities are unknown. This is explicitly mentioned where relevant. |
| Reproducibility | We report observational findings, no experimental manipulations were taken place. |
| Randomization | All individuals were measured simultaneously, so this does not apply. |

| | |
|---|---|
| Blinding | Different persons have prepared the data acquisition (where recognising the individuals was essential) and the data analysis. During the data analysis automatically given tags were used as identifiers, which were only replaced by the identity of the individuals later. At the final stage of the analysis, identities were visible for all authors. |

Did the study involve field work? ☒ Yes ☐ No

## Field work, collection and transport

| | |
|---|---|
| Field conditions | The aerial observations were carried out on dry, summer days (August 17, August 24, September 5, September 13, and October 2 in 2018), during daylight hours (between 8am – 3pm local time) with temperature ranging between 20 and 32 °C. The wind was below 7m/s. |
| Location | 47°31'3.3"N 21°5'34.1"E at Pentezug Reserve, Hortobagy National Park, Hungary |
| Access & import/export | The data acquisition was done by employees of the Hortobagy National Park Directorate or with their approval. Pentezug Reserve is a strictly protected area. The research was approved by the Government Office for Hajdú-Bihar County (Hungary) under the reference number HB-03/KTF/00779-24/2017, and by the Hortobágy National Park Directorate under the reference number 3482-2/2017. |
| Disturbance | The aerial observation using drones may disturb the animals. Prior to the study we tested the disturbance of drones with decreasing flying altitudes and observed that horses started to avoid drones at around 3-4m flying altitudes. To minimise disturbance, we did not fly the drones lower than 10m (as a conservative approach). The horses were approach by the observers on foot, parking the car no closer than 200m from the herd. |

# Reporting for specific materials, systems and methods

We require information from authors about some types of materials, experimental systems and methods used in many studies. Here, indicate whether each material, system or method listed is relevant to your study. If you are not sure if a list item applies to your research, read the appropriate section before selecting a response.

### Materials & experimental systems

| n/a | Involved in the study | |
|---|---|---|
| ☒ | ☐ | Antibodies |
| ☒ | ☐ | Eukaryotic cell lines |
| ☒ | ☐ | Palaeontology and archaeology |
| ☐ | ☒ | Animals and other organisms |
| ☒ | ☐ | Clinical data |
| ☒ | ☐ | Dual use research of concern |

### Methods

| n/a | Involved in the study | |
|---|---|---|
| ☒ | ☐ | ChIP-seq |
| ☒ | ☐ | Flow cytometry |
| ☒ | ☐ | MRI-based neuroimaging |

## Animals and other research organisms

Policy information about studies involving animals; ARRIVE guidelines recommended for reporting animal research, and Sex and Gender in Research

| | |
|---|---|
| Laboratory animals | This study did not involve laboratory animals. |
| Wild animals | Przewalski's horses (Equus ferus przewalskii) were observed in the field. All animals of the population were used in this study, their age ranged between 0 to 23 years old. Animals were not caught for the study. Genetic sampling were done as a standard procedure by National Park for species conservation purposes and the data was provided by them for this research. |
| Reporting on sex | Sex of the individuals were identified by visual observation and backed by genetic sampling. All individuals of the population were used in this study, 330 females, 326 males and 656 in total. Analysis were performed separately for female and male horses. |
| Field-collected samples | This study did not involve samples collected from the field for our research. |
| Ethics oversight | The research was approved by the Government Office for Hajdú-Bihar County (Hungary) under the reference number HB-03/KTF/00779-24/2017, and by the Hortobágy National Park Directorate under the reference number 3482-2/2017. |

Note that full information on the approval of the study protocol must also be provided in the manuscript.
